# Targeting ligament boundaries rather than alignment boundaries in augmented‐reality‐assisted total knee arthroplasty ensures comparable gait patterns between operated and contralateral limbs

**DOI:** 10.1002/ksa.70312

**Published:** 2026-01-28

**Authors:** Michael Engl, Fjorela Qordja, Guido Bocchino, Giulio Maccauro, Sebastian Schaller, Andrea Giordano Salvi, Luca De Berardinis, Michele Paolo Festini Capello, Pieralberto Valpiana, Pier Francesco Indelli

**Affiliations:** ^1^ Department Orthopaedic Surgery Südtiroler Sanitätsbetrieb Brixen Italy; ^2^ Institute for Biomedicine, EURAC Institute Bozen Italy; ^3^ Personalized Arthroplasty Society (PAS), One Glenlake Parkway NE, Suite 1200 Atlanta Georgia USA; ^4^ Department of Clinical and Molecular Sciences Università Politecnica delle Marche, Clinic of Orthopaedics Ancona Italy; ^5^ Institute of Biomechanics Paracelsus Medical University (PMU) Salzburg Austria; ^6^ Department of Aging, Neurological, Orthopaedic and Head‐Neck Sciences Fondazione Policlinico Universitario Agostino Gemelli IRCCS Rome Italy; ^7^ Orthopaedic Department IRCCS Ospedale San Raffaele Milan Italy; ^8^ The Breyer Center for Overseas Studies in Florence Stanford University Florence Italy

**Keywords:** alignment, augmented reality, balancing, enabling technologies, knee, total knee arthroplasty

## Abstract

**Purpose:**

Modern personalised total knee arthroplasty (TKA) techniques aim to mimic natural knee kinematics by focusing on soft‐tissue dynamics rather than static alignment. However, a consistent definition of the optimal ligamentous balance has not yet been established. This study had three aims: to assess intraoperative collateral ligament tension and the overall balance between the medial and lateral compartments; to evaluate postoperative gait analysis parameters and compare them with the healthy contralateral knee; and to determine whether the achieved intercompartmental balance affects the final coronal alignment of the knee.

**Methods:**

A prospective single‐cohort study was conducted to evaluate 102 primary AR‐assisted TKAs performed for end‐stage unilateral knee osteoarthritis between September 2021 and June 2024. A total of 17 patients out of 102 were ultimately included following the application of the study's inclusion and exclusion criteria. Intraoperative data were collected on MCL and LCL elongation patterns during passive ROM both before and after trial component placement. Additionally, the study examined differences in intercompartmental ligament balancing. Postoperative coronal alignment was radiographically evaluated using the hip‐knee‐ankle (HKA) angle. Functional outcomes were measured using 3D markerless gait analysis at least 9 months after surgery. A repeated‐measures analysis of variance (ANOVA) and a Pearson correlation were employed to examine trends in ligament asymmetry and their relationship with alignment.

**Results:**

The medial collateral ligament (MCL) exhibited nearly isometric behaviour throughout the range of motion (0.3 ± 1.11 at 0°, −2.4 ± 2.71 at 20°, −2.3 ± 3.96 at 40°, −2.3 ± 4.16 at 60°, −1.5 ± 4.03 at 80°, −0.7 ± 4.52 at 100°, −0.2 ± 4.97 at 120° preoperatively; 2.2 ± 2.98 at 0°, 2.1 ± 3.40 at 20°, 1.4 ± 4.02 at 40°, 1.2 ± 4.41 at 60°, 0.8 ± 4.48 at 80°, 1.5 ± 4.42 at 100°, 1.6 ± 5.09 at 120° postoperatively). Conversely, the LCL showed progressive detensioning (0.0 ± 2.03 at 0°, −3.1 ± 2.07 at 20°, −3.9 ± 2.72 at 40°, −4.9 ± 3.34 at 60°, −6.6 ± 3.63 at 80°, −8.5 ± 4.13 at 100°, −9.6 ± 4.40 at 120° preoperatively; −2.1 ± 5.16 at 0°, −3.1 ± 4.03 at 20°, −4.7 ± 3.80 at 40°, −5.8 ± 3.19 at 60°, −7.0 ± 3.86 at 80°, −6.6 ± 4.55 at 100°, −7.0 ± 5.08 at 120° postoperatively), leading to asymmetric ligament balancing with the opening of the lateral compartment. This trapezoidal soft‐tissue configuration persisted postoperatively. No significant differences were observed in gait parameters between the operated and contralateral limbs, all *p*‐values being > 0.05. A notable negative correlation was seen between postoperative HKA and ligament asymmetry at early flexion angles (0°, 20°, 40°; *r* = −0.63, *r* = −0.67, *r* = −0.48, respectively), indicating that soft‐tissue balancing affects coronal alignment.

**Conclusions:**

Focusing on dynamic soft‐tissue boundaries rather than static alignment targets may preserve knee kinematics after TKA. Augmented reality‐assisted balancing techniques provide real‐time, personalised ligament management, which aligns with the patient's native kinematics and could be an encouraging direction in modern knee arthroplasty. More research is needed to confirm these findings across larger groups and different technologies.

**Level of Evidence:**

Level III.

**Abbreviations**
AR: augmented reality
BMI: body mass index
CPAK: coronal plane alignment of the knee
CT: computer tomography
HKA: hip‐knee‐ankle
IRB: Institutional Review Board
KA: kinematic alignment
LCL: lateral collateral ligament
MA: mechanical alignment
MC: medial congruent
MCL: medial collateral ligament
OA: osteoarthritis
ROM: range of motion
SD: standard deviation
TKA: total knee arthroplasty

## INTRODUCTION

Total knee arthroplasty (TKA) is increasingly evolving towards personalised procedures that incorporate knee phenotypes into surgical planning [20]. The potential benefit of this personalisation is its capacity to restore natural knee kinematics more effectively than a standard, one‐size‐fits‐all approach [18]. To accurately replicate the knee phenotype, various classifications have been used [15, 24], all of which refer to anatomical landmarks identified on static, full‐length leg radiographs (LLRs) or computed tomography (CT) scans. Unfortunately, many of these classifications overlook a crucial biomechanical parameter of knee motion: the viscoelastic behaviour and tension of peri‐articular soft‐tissue. From a purely biomechanical perspective, joint laxity refers to intra‐articular displacements and rotations that occur in response to applied forces or moments [39]. Since the 1990s, dedicated knee simulators have been developed to assess cadaveric specimens and TKA constructs, aiming to standardise and measure the viscoelastic behaviour of the knee joint. The advent of enabling technologies has enhanced the surgeon's ability to quantify native knee laxity, including the excessive or reduced laxity patterns observed in extreme phenotypic variants during TKA [10, 11].

However, because there is no clear definition of safe soft‐tissue boundaries in knee arthroplasty, it ultimately falls to the surgeon to decide whether to accept or adjust knee laxity during TKA. Although there is no clear consensus on safe limits for knee laxity, a commonly accepted goal is to replicate the isometric behaviour of the medial collateral ligament (MCL) [38]. Current research lacks data linking intraoperative measurements of MCL and lateral collateral ligament (LCL) elongation with objective postoperative kinematic outcomes, limiting our understanding of how different balancing strategies affect function. To address this gap, a few of the available augmented reality (AR) systems [30, 34] can restore both the native joint line orientation and natural knee laxity through accurate soft‐tissue tensioning, aiming to replicate more natural knee motion.

The primary aim of this study was to assess ligament behaviour throughout the knee's flexion–extension range, focusing on the tension and relaxation of the individual MCL and LCL, as well as the overall balance between the medial and lateral compartments. The second objective was to evaluate patient outcomes using objective gait analysis data and to compare them with those of the healthy contralateral knee. The third goal was to determine whether the achieved intercompartmental balance was associated with the final coronal alignment of the knee. The authors hypothesised different ligamentous behaviours, with no differences in gait analysis parameters between the surgical and healthy contralateral knees, and no correlation with the final hip‐knee‐ankle (HKA) axis.

## MATERIAL AND METHOD

### Study design and setting

A prospective single‐cohort study was conducted at our institution to evaluate 17 patients among 102 consecutive TKAs performed for end‐stage unilateral knee osteoarthritis (OA) between September 2021 and June 2024. All procedures used the same medial pivot GMK Sphere implant (Medacta International SA) and were assisted by the NextAR system.

### Inclusion and exclusion criteria

The study's inclusion criteria were: age between 18 and 80 years; isolated end‐stage knee OA; healthy contralateral knee with Kellgren‐Lawrence 0 or maximum 1; minimum follow‐up of 9 months; absence of musculoskeletal or neurological disorders affecting the lower limbs; no previous surgeries on the knees, hips, ankles, feet or spine; no radiographic posttraumatic, lower limb deformities. Varus (HKA < 177°) or neutral knees (177° ≤ dHKA ≤ 183°) [2, 29] were included in the analysis. Conversely, patients were excluded if they experienced major postoperative complications (such as infections and revision surgeries), had advanced radiographic OA or referred pain in the contralateral knee or any lower extremity joint, or were unwilling to participate in the study.

After the telephone interview and applying the criteria above, only patients who met the patient‐acceptable symptom state (PASS) thresholds for functional outcome scores were considered eligible for gait analysis. Specifically, inclusion required an Oxford Knee Score of at least 32 [5, 6] and a Forgotten Joint Score‐12 of at least 77 [33].

### Surgical technique

All patients underwent a preoperative hip, knee and ankle CT to determine the patient phenotype and the static alignment of the lower limb. Preoperative planning was performed before surgery, utilising restricted kinematic alignment (rKA) principles to establish alignment boundaries within ±5° of an HKA angle, a modification of the original technique [30].

This planning has always been considered preliminary, as the final alignment was determined intraoperatively based on the tension of the MCL and LCL. The AR system used in this study (comprising a pair of smart glasses, two small single‐use wireless sensors attached to the femur and tibia with pins, and a control unit) enables intraoperative, dynamic landmarking of the knee after standard capsulotomy. This process includes not only the planning of bone cuts and component positioning but also monitoring the intraoperative behaviour of the MCL and LCL during passive range of motion (ROM) as part of the planning. Varus/valgus stress across the entire ROM was applied for the AR system to define the limits of maximum elongation and shortening of the collateral ligaments. The final planning included, first, reproducing a slight ligament asymmetry (aiming for MCL isometry and slight LCL de‐tension during ROM) and second, an alignment based on rKA principles. Once the knee was evaluated for residual laxity, the pins and trial components were removed and the final components were cemented into place.

### Demographic and radiographic data

Demographic variables, including age, sex, body mass index (BMI) and operated side, were recorded. The pre and postoperative HKA measured on LLRs were analysed. The mean preoperative HKA was classified according to the coronal plane alignment of the knee (CPAK) classification [24].

### Intraoperative ligamentous data

The ligament data for both the MCL and LCL were expressed as normalised, dimensionless values relative to a subject‐specific length (*L*_₀_). The reference length *L*₀ was predicted preoperatively using CT‐derived three‐dimensional anatomical modelling from the NextAR system. Ligament length variations were therefore reported as relative changes rather than absolute millimetre values to reduce intersubject variability. The system provides measurements with a resolution of 0.1 units. All quantitative analyses were performed under passive conditions, with the knee allowed to flex and extend under gravity, without varus or valgus stress.

The intraoperative tension and detension behaviour of the MCL and LCL were expressed as elongation values (when positive) or shortening values (when negative) relative to the baseline length. In the present study, when a ligament had negative values, it indicated that the ligament had de‐tensioned, and therefore, the corresponding compartment had opened. Conversely, when a ligament showed positive values, it meant that the ligament maintained or increased its tension, keeping the corresponding knee compartment closed.

All ligament measurements were recorded both at the end of intraoperative planning (preop) and after placing the trial components (postop). The intercompartmental ligamentous balancing (Δ) was calculated as the difference between the behaviours of the MCL and LCL at all knee flexion‐extension angles (Δ ligamentous balancing _θ_ = MCL _θ_ – LCL _θ_).

### Gait analysis

Formal gait analysis was conducted at least 9 months after surgery. We used a modern, markerless instrumented treadmill (Walkerviewer—WV by TecnoBody, Italy) equipped with an instrumented belt embedded with eight load cells, a 48‐inch wide liquid crystal display screen providing continuous virtual reality and biofeedback, a 3D camera for motion capture (Kinect v2, Microsoft) and a 15‐inch touchscreen connected to a personal computer.

The WV‐integrated software allowed the collection of spatiotemporal parameters, including walking speed, cadence, step length (for both the operated and nonoperated limbs), symmetry and contact time per limb. ROM at the hip and knee was recorded for both limbs.

Postural control was evaluated by analysing trunk motion in both the sagittal (flexion/extension) and frontal (lateral bending) planes, as well as the displacement of the centre of gravity (COG), measured in centimetres and as a percentage of the gait cycle. Specific knee joint kinematic parameters were obtained from the gait knee graphs using PlotDigitizer Software. In particular, the following parameters were considered: knee flexion at heel strike, knee maximum flexion during the stance phase, knee maximum extension during the stance phase, knee ROM amplitude during the stance and knee maximum flexion during the swing phase.

Parameters of the surgical limb were compared to those of the contralateral, healthy knee.

### Statistical analysis

Data were collected and organised using Microsoft Excel (Microsoft Corp.). Statistical analysis was performed with SPSS 26 (IBM Corp.). The normality of the continuous variables was confirmed using the Shapiro–Wilk test. Continuous variables are reported as mean ± standard deviation (SD), while categorical variables are presented as absolute frequencies and percentages. The tension and detention behaviour of the MCL and LCL during knee motion, both before and after surgery, was evaluated using paired *t*‐tests comparing each flexion angle to the baseline at 0°. Bonferroni correction was applied to reduce type I error. A repeated measures analysis of variance (ANOVA), followed by Bonferroni analysis, was conducted to assess the significance of the Δ ligamentous balancing across the full range of knee flexion, both pre and postoperatively.

Regarding gait data, a paired *t*‐test was performed to assess the significance of the difference between the surgical and healthy knees. Pearson correlation coefficients were calculated between the postoperative HKA angle measured on LLRs and the postoperative Δ ligamentous balancing _θ_ at each degree of ROM to investigate potential links between final coronal alignment and compartmental soft‐tissue balancing. Significance was set at an alpha value of 0.05. An a priori power analysis was performed assuming a two‐tailed paired design, a statistical power of 80%, an alpha error probability of 0.05 and a calculated effect size of 0.72. The calculated effect size was deemed appropriate based on previous gait analysis literature reporting moderate to large standardised effects for gait‐related outcomes after total knee replacement [25], with effect sizes ranging from approximately 0.33 to 0.89 [27]. Based on these assumptions, the minimum required sample size was 16 subjects.

## RESULTS

Seventeen patients were ultimately enrolled following the study's inclusion and exclusion criteria.

The eligibility process is also illustrated in Figure 1.

**Figure 1:**
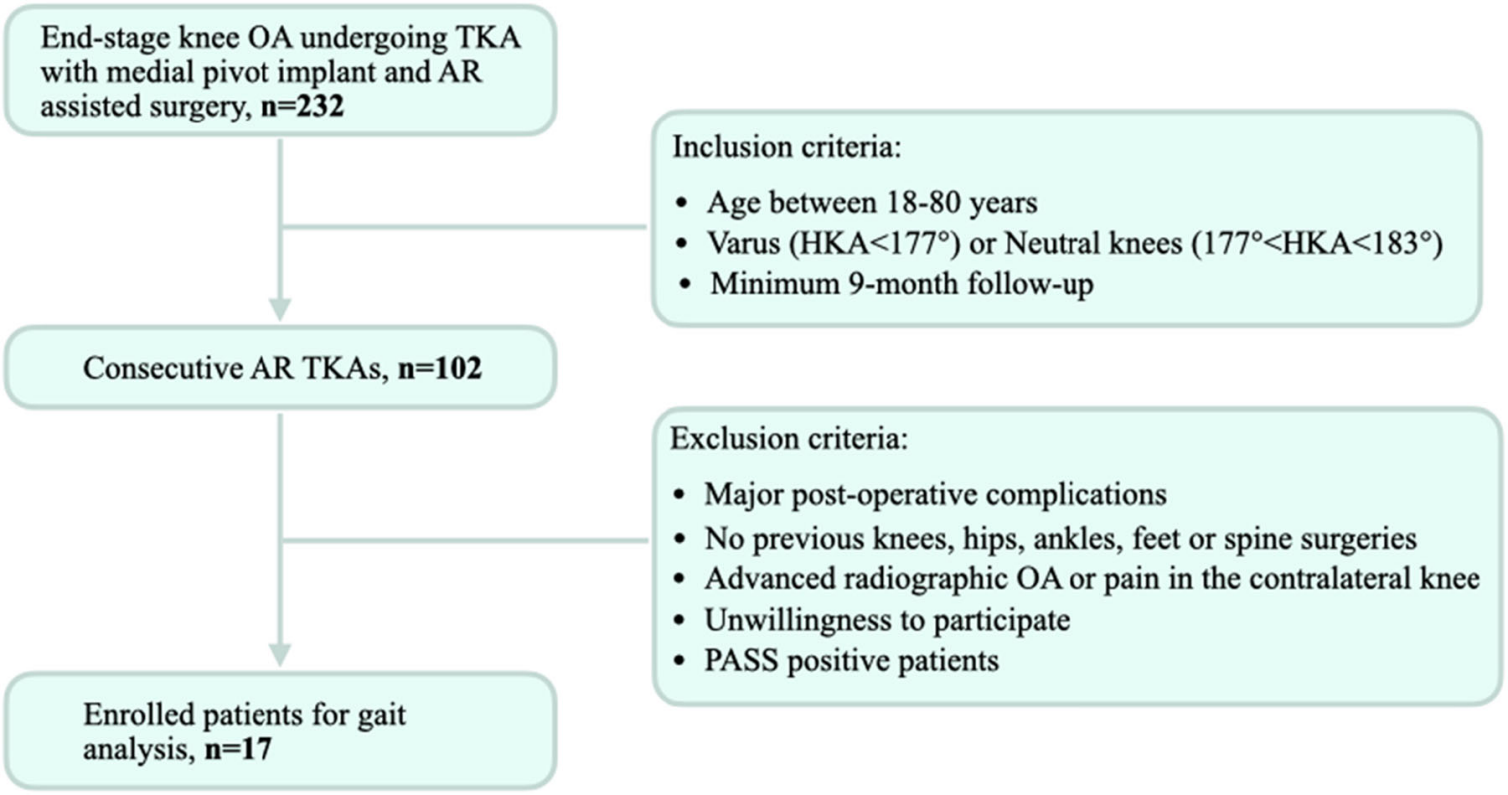
Patients' eligibility criteria. AR, augmented reality; HKA, hip‐knee‐ankle; OA, osteoarthritis; TKA, total knee arthroplasty.

Among them, 8 (47.1%) were male and 9 (52.9%) were female. The left knee was involved in nine cases (52.9%), while the remaining eight cases (47.1%) involved the right knee. The average age at surgery was 69 ± 9.04 years, with a mean BMI of 28.22 ± 5.13.

### MCL and LCL dynamic behaviour

MCL's tension and detention behaviour are shown as average values in Table 1 and are depicted in Figure 2. Positive numbers indicate MCL stretching beyond full extension (0°), signifying the medial compartment remains tight, while negative numbers suggest ligament de‐tensioning.

**Table 1:** MCL values and statistical differences through the range of motion.

Degrees of flexion	MCL			
	Preop behaviour (Mean ± SD)	*p*‐Value	Postop behaviour (Mean ± SD)	*p*‐Value
0°	0.3 ± 1.11	‐	2.2 ± 2.98	‐
20°	−2.4 ± 2.71	**<0.008**	2.1 ± 3.40	0.794
40°	−2.3 ± 3.96	0.008	1.4 ± 4.02	0.174
60°	−2.3 ± 4.16	0.013	1.2 ± 4.41	0.158
80°	−1.5 ± 4.03	0.053	0.8 ± 4.48	0.142
100°	−0.7 ± 4.52	0.303	1.5 ± 4.42	0.446
120°	−0.2 ± 4.97	0.607	1.6 ± 5.09	0.587

*Note*: In bold significant values according to Bonferroni when compared to 0°. Abbreviations: MCL, medial collateral ligament; SD, standard deviation.

**Figure 2:**
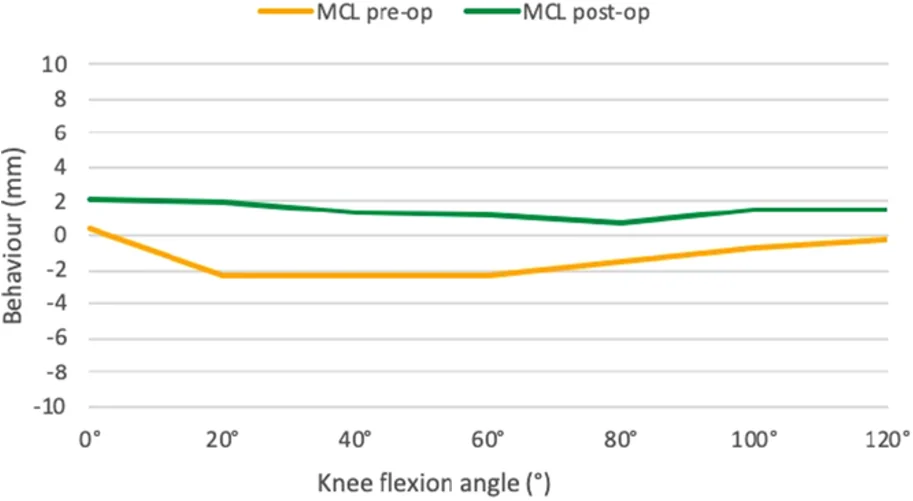
MCL's close‐to‐isometry behaviour during knee flexion. MCL, medial collateral ligament.

According to the paired *t*‐test, the dynamic pattern of the MCL did not show statistically significant differences at any flexion angle compared to baseline (0°), either before or after the operation, except for the preoperative measurement at 20° (Table 1).

The MCL tension/de‐tension trend during ROM exhibits a behaviour that is close to isometric.

LCL's tension and detention behaviours are shown as average values in Table 2 and are depicted in Figure 3. Positive values represent an increase in LCL tension relative to full extension (0°), whereas negative values indicate detensioning of LCL fibres, which corresponds to opening of the compartment. Paired *t*‐test analysis showed that the progressive shortening of the LCL at all flexion angles, compared to baseline (0°), was consistently statistically significant, except for the postoperative measurement at 20° (Table 2). The LCL trend during ROM indicated increasing laxity.

**Table 2:** LCL values and statistical differences through the range of motion.

Degrees of flexion	LCL			
	Preop behaviour	*p*‐Value	Postop behaviour	*p*‐Value
	Mean ± SD		Mean ± SD	
0°	0.0 ± 2.03	‐	−2.1 ± 5.16	‐
20°	−3.1 ± 2.07	**<0.008**	−3.1 ± 4.03	0.109
40°	−3.9 ± 2.72	**<0.008**	−4.7 ± 3.80	**<0.008**
60°	−4.9 ± 3.34	**<0.008**	−5.8 ± 3.19	**<0.008**
80°	−6.6 ± 3.63	**<0.008**	−7.0 ± 3.86	**<0.008**
100°	−8.5 ± 4.13	**<0.008**	−6.6 ± 4.55	**<0.008**
120°	−9.6 ± 4.40	**<0.008**	−7.0 ± 5.08	**<0.008**

*Note*: In bold significant values according to Bonferroni when compared to the 0°. Abbreviations: LCL, lateral collateral ligament; SD, standard deviation.

**Figure 3:**
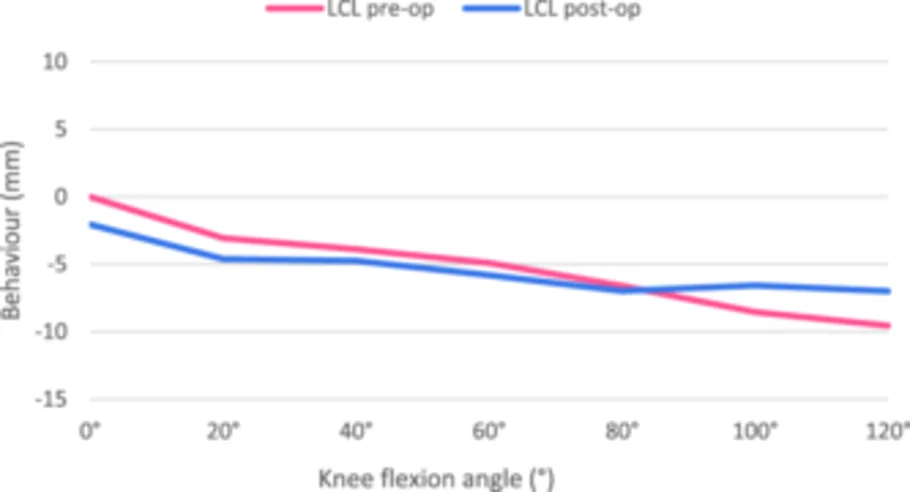
LCL's progressive laxity during knee flexion. LCL, lateral collateral ligament.

### Asymmetric ligament balancing

The mean Δ ligamentous balancing was calculated for each degree of flexion‐extension (both pre and postoperatively); results are shown in Table 3.

**Table 3:** Asymmetric ligament balance during the arc of motion.

Degrees of flexion	Preop Δ ligamentous balancing (mean ± SD)	Postop Δ ligamentous balancing (mean ± SD)
0°	0.4 ± 1.97	4.2 ± 6.39
20°	0.7 ± 3.49	5.1 ± 5.80
40°	1.6 ± 4.71	6.1 ± 5.20
60°	2.6 ± 4.74	7.0 ± 4.95
80°	5.1 ± 4.54	7.8 ± 4.86
100°	7.8 ± 5.32	8.0 ± 5.65
120°	9.3 ± 5.51	8.6 ± 6.02

Abbreviation: SD, standard deviation.

The ligamentous balancing between the MCL and LCL was always asymmetric, with the MCL's elongation consistently being less than the increase in laxity of the LCL. This created an asymmetric ligament balance, characterised by constant tension in the MCL and increasing laxity in the LCL, leading to a greater opening of the lateral compartment. The resulting trapezoidal asymmetric ligament behaviour is shown in Figure 4.

**Figure 4:**
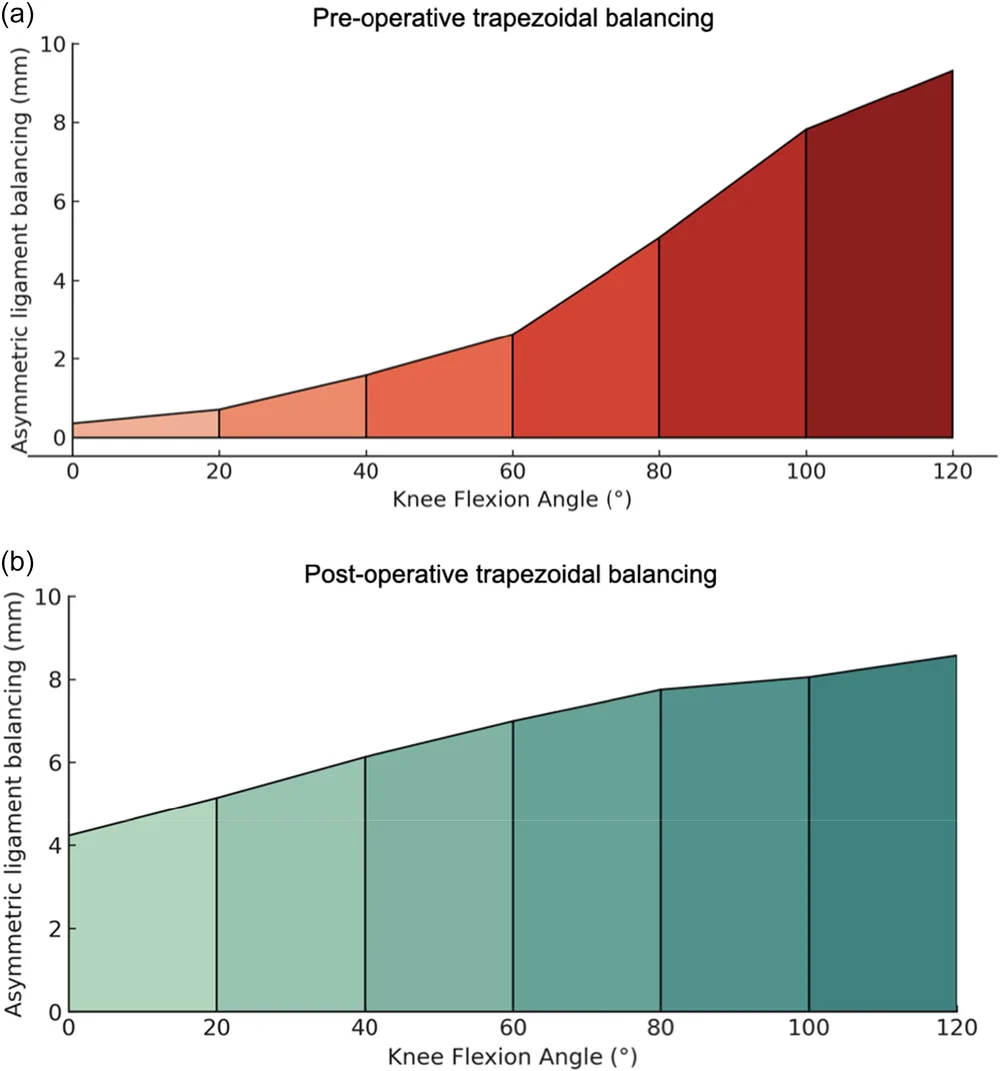
Pre and postoperative asymmetric ligament balancing.

Preoperatively, the repeated measures ANOVA showed a statistically significant effect of flexion angle on ligament balance asymmetry (*p* < 0.001). Post hoc pairwise comparisons with Bonferroni correction indicated that asymmetry increased significantly at higher flexion angles compared to full extension. The postoperative ANOVA did not reveal significant differences in intercompartmental ligament asymmetry across flexion angles (*p* = 0.222).

### Gait analysis results

Gait analysis data collected after the asymmetrical ligament balancing of the TKAs are reported in Table 4. When compared to the contralateral healthy knee, no statistical differences were found for both kinematic and spatiotemporal gait parameters.

**Table 4:** Gait analysis results.

Gait parameters	General	Surgery side	Healthy side	
	Mean ± SD	Mean ± SD	Mean ± SD	*p*‐Value
Trunk flexion/extension (°)	11.0 ± 4.76			
Trunk lateral flexion (°)	−0.4 ± 1.42			
Hip ROM (°)		33.1 ± 4.21	32 ± 3.72	0.288
Knee ROM (°)		49.0 ± 9.34	48.6 ± 8.24	0.837
Symmetry (%)	2.8 ± 2.72			
Walking speed (Km/h)	2.0 ± 0.62			
Cadence (cycle/s)	0.8 ± 0.15			
Step length (cm)		36.6 ± 8.87	37.6 ± 9.23	0.341
Step_length (%)		7.8 ± 3.62	8.2 ± 5.29	0.728
Contact time (s)		1.0 ± 0.25	1.0 ± 0.26	0.625
COG vertical oscillation (cm)	1.7 ± 0.58			
COG vertical oscillation (%)	1.9 ± 0.59			
Heel strike (°)		10.3 ± 8.78	8.5 ± 8.31	0.135
Stance max flexion (°)		9.3 ± 5.99	8.7 ± 6.42	0.524
Stance max extension (°)		5.1 ± 5.89	3.7 ± 3.19	0.309
Stance ROM amplitude (°)		7.4 ± 7.10	7.2 ± 7.38	0.889
Swing max flexion (°)		52.6 ± 7.68	50.5 ± 9.06	0.249

Abbreviations: COG, centre of gravity; ROM, range of motion; SD, standard deviation.

### Radiological assessment

The mean preoperative HKA was 177.6 ± 3.96, with 15 out of 17 knees classified as CPAK I and the remaining two as CPAK III. The cohort mean postoperative HKA increased to 178.9 ± 3.50. Pearson correlation analysis confirmed a significant negative relationship between postoperative HKA and the postoperative asymmetric ligament balancing at 0°, 20° and 40° (*r*[15] = –0.63, *p* = 0.007; *r*[15]) = –0.67, *p* = 0.004; *r*[15] = –0.48, *p* = 0.049, respectively), with lower postoperative HKA values (i.e., more varus alignment) being associated with greater postoperative ligament asymmetry. Indeed, negative correlations at higher flexion angles remained weaker and nonsignificant.

## DISCUSSION

The most important findings of this study were derived from a basic science assessment of an AR‐assisted TKA surgical technique focused on reproducing an asymmetric ligamentous balance, characterised by near‐isometric MCL behaviour during flexion and increasing LCL laxity. When the TKA was balanced according to this ligamentous asymmetry, gait analysis showed no statistically significant differences compared with the contralateral healthy knee, strongly supporting the functional validity of this targeted, phenotype‐driven tensioning strategy. Notably, the observed ligamentous asymmetry showed a significant negative correlation with postoperative coronal alignment (HKA) up to 60° of flexion, suggesting an impact on the final alignment outcome.

The use of enabling technologies in TKA is shifting the focus of surgical technique from static alignment‐based planning to real‐time functional personalisation [14, 19]. While preoperative imaging defines bone morphology and phenotype, it provides limited information about soft‐tissue dynamics [31, 32].

Intraoperative tools, such as AR systems like NextAR (Medacta, SA), now allow for direct measurement of ligament behaviour, enabling truly personalised balancing strategies based on real‐time data [1, 37]. In this study, AR technology enabled precise measurement of the MCL's near‐isometric behaviour, as its length (and thus tension) remained consistent throughout the ROM, both before and after implant placement, except at the preoperative measurement at 20°. This finding is consistent with previous kinematic studies, which indicated that the fibres of the MCL experience minimal length changes during ROM [8, 13, 23, 38].

Hosseini Nasab et al. [17] specifically examined 3D in vivo joint kinematics using dynamic fluoroscopy and found that none of the ligament bundles showed purely isometric behaviour during the activities studied across any of the implant designs. Conversely, our results indicated that the LCL gradually loses tension during flexion, leading to opening of the lateral compartment. This observation aligns with previous reports in the literature, which have noted increased laxity and shortening of the LCL fibres as knee flexion progresses [4, 7].

Unlike the present study, a previous cadaveric study by Belvedere et al. [3] showed that the MCL and LCL fibres reached their maximum length in full knee extension and gradually shortened during the flexion arc, indicating that these ligamentous structures slackened progressively during passive knee flexion [3]. Given this premise, accurate soft‐tissue balancing in TKA is crucial, as variations in ligament tension can cause instability [40].

Indeed, orthopaedic surgeons understand that TKA failure is mainly due to infection, instability, loosening and stiffness [38], rather than to ‘malalignment’ relative to the original knee phenotype [13]. Furthermore, a linear relationship has been established between ligament tension and tibiofemoral contact forces, suggesting that ligaments may account for up to 74%–80% of joint loading during flexion. Verstraete et al. [36] measured contact forces in the tibiofemoral joint and found that the medial compartment consistently bears higher loads than the lateral one (114 N vs. 63 N at 0° flexion; 30 N vs. 6 N at 90° flexion), supporting the idea of asymmetric load distribution.

Examining the intercompartmental ligamentous balance, our cohort showed a notably asymmetric ligament balance, with constant tension in the MCL and increasing laxity in the LCL. As a result, we observed greater opening of the lateral compartment, creating a trapezoidal space and asymmetric ligament behaviour. First, this approach aligns with the idea of an ‘asymmetric gap’ in the coronal and sagittal planes, where physiological laxity gradients are preserved rather than evened out. Second, this ligament asymmetry reflects the native knee's biomechanics, enabling the replication of physiological laxity [35].

This typical MCL/LCL configuration has been identified as a key factor influencing lateral pivot behaviour during stance and medial pivot kinematics in the swing phase of gait [9, 12, 22]. Our findings support this model by demonstrating that maintaining natural tension asymmetry is associated with preserved gait characteristics and a gait pattern comparable to that of the contralateral healthy limb. Notably, no significant differences were observed in kinematic or spatiotemporal gait parameters: the prosthetic knee with the ligamentous balancing approach performed similarly to the contralateral healthy limb. Furthermore, this asymmetric functional behaviour underpins the biomechanical basis for medial pivot kinematics. Hodgeson et al. [16] reported that medial pivot and medially congruent TKA designs simulate natural knee motion by establishing a stable medial centre of rotation and a more mobile lateral compartment that follows an arcuate path.

Interestingly, our cohort's preoperative asymmetric ligament balancing showed considerable variability, highlighting the unstable, pathological nature of asymmetry typical of arthritic knees. Postoperatively, although asymmetry persisted, it became more consistent and no longer statistically significant, suggesting a shift towards more normal ligament behaviour after implantation.

Finally, ligament asymmetry showed a significant negative correlation with HKA up to 60° of knee flexion. This indicates that, during early flexion, variations in coronal alignment are associated with changes in ligament balance, likely reflecting the predominant role of passive stabilisers, particularly the collateral ligaments. Beyond 60° of flexion, this relationship was no longer observed, suggesting that additional factors, such as dynamic stabilisers and implant‐related kinematics, may increasingly contribute to knee behaviour at higher flexion angles.

These findings align with a previous study in which we observed a progressive varus trend in the HKA up to 60°, followed by partial recovery that did not fully restore neutral alignment [28]. Interestingly, another recently published cross‐sectional study [26] established a correlation between the coronal axis and ligament behaviour: the authors noted a progressive shift of a tight MCL in flexion in varus phenotypes towards loosening of the MCL in valgus phenotypes, making the arithmetic HKA a reliable predictor of MCL behaviour across the ROM.

Additionally, a recent study by Graichen et al. [10] examined the relationship between coronal laxity and HKA angle, analysing medial‐lateral laxity patterns during both extension and flexion under different alignment strategies. Using a personalised bony phenotype approach, they found only a weak correlation between HKA and laxity in extension and no correlation in flexion. In contrast, a standard mechanical alignment method showed a strong correlation in extension and more consistent laxity patterns in flexion. However, the current study assessed ligament behaviour dynamically across the full ROM, measuring the uneven balance between the MCL and LCL. This dynamic, function‐focused assessment differs from a static, gap‐based method and suggests that individualised ligament balancing could have a more direct and measurable impact on coronal alignment than models based solely on bony structures have indicated [21].

This study has several significant limitations. First, the patient cohort is small due to strict inclusion criteria, and not all CPAK phenotypes are represented. The authors included only patients with isolated knee OA to prevent involvement of other joints (hips, knees, ankles, spine) from affecting gait analysis outcomes. All included patients met the established PASS thresholds for functional outcome scores, ensuring a homogeneous cohort with respect to clinical status at follow‐up. Second, intraoperative data collection was operator‐dependent, potentially leading to variability in ligament elongation measurements.

Third, the extensor mechanism was left open during intraoperative measurements because tracker pins were present within the joint; this could have influenced soft‐tissue tension during recording. Additionally, the gait analysis platform used in this study could not obtain 3D data, so the impact of knee axial rotation during gait phases could not be assessed. Finally, only one image‐based AR system has been studied, indicating that the current results might not apply to other AR systems. Future research should explore whether these findings can be reproduced using different enabling technologies and larger patient groups.

## CONCLUSION

In this study, the target of ligament asymmetry throughout the ROM was associated with comparable gait kinematics between the operated and contralateral healthy knees. These findings suggest that intraoperative evaluation of soft‐tissue behaviour across knee flexion may complement alignment‐based approaches in personalised TKA and warrant further investigation in larger, dedicated cohorts.

## AUTHOR CONTRIBUTIONS

All authors contributed to the conception and design of the study. Michael Engl, Pier Francesco Indelli, Pieralberto Valpiana, Fjorela Qordja, Christian Coppola and Guido Bocchino participated in data collection. Luca De Berardinis, Fjorela Qordja and Andrea Giordano Salvi participated in the statistical analysis. Pieralberto Valpiana, Guido Bocchino, Andrea Salvi, Sebastian Schaller and Fjorela Qordja participated in the interpretation of the data. Guido Bocchino and Fjorela Qordja drafted the work. Pier Francesco Indelli and Giulio Maccauro revised the manuscript and approved the version to be published. All authors reviewed and approved the final version of the manuscript.

## CONFLICT OF INTEREST STATEMENT

Pier Francesco Indelli is a consultant for medical education for Zimmer Biomet, Warsaw, USA and MicroPort, China. The remaining authors declare no conflicts of interest regarding the manuscript's content.

## ETHICS STATEMENT

This study was conducted following the principles of the Declaration of Helsinki. This study received approval from the Internal Review Board (IRB: SABES 71/2023 and 17/2024). Written consent was obtained from participants in the study.

## Data Availability

The datasets used and/or analysed during this study are available from the corresponding author upon reasonable request.

## References

[1] Ascani D , Mazzà C , De Lollis A , Bernardoni M , Viceconti M . A procedure to estimate the origins and the insertions of the knee ligaments from computed tomography images. J Biomech. 2015;48(2):233–237. 10.1016/j.jbiomech.2014.11.041 25512017

[2] Bellemans J , Colyn W , Vandenneucker H , Victor J . The Chitranjan Ranawat award: is neutral mechanical alignment normal for all patients?: the concept of constitutional varus. Clin Orthop Relat Res. 2012;470(1):45–53. 10.1007/s11999-011-1936-5 PMC3237976 21656315

[3] Belvedere C , Ensini A , Feliciangeli A , Cenni F , D'Angeli V , Giannini S , et al. Geometrical changes of knee ligaments and patellar tendon during passive flexion. J Biomech. 2012;45(11):1886–1892. 10.1016/j.jbiomech.2012.05.029 22677336

[4] Clément J , Blakeney W , Hagemeister N , Desmeules F , Mezghani N , Lowry V , et al. Hip‐knee‐ankle (HKA) angle modification during gait in healthy subjects. Gait Posture. 2019;72:62–68. 10.1016/j.gaitpost.2019.05.025 31151089

[5] Clement ND , MacDonald D , Simpson AHRW . The minimal clinically important difference in the Oxford knee score and Short Form 12 score after total knee arthroplasty. Knee Surg Sports Traumatol Arthrosc. 2014;22(8):1933–1939. 10.1007/s00167-013-2776-5 24253376

[6] Conner‐Spady BL , Marshall DA , Bohm E , Dunbar MJ , Loucks L , Noseworthy TW . Patient acceptable symptom state (PASS): thresholds for the EQ‐5D‐5L and Oxford hip and knee scores for patients with total hip and knee replacement. Qual Life Res. 2023;32(2):519–530. 10.1007/s11136-022-03287-9 36367656

[7] Deep K , Eachempati KK , Apsingi S . The dynamic nature of alignment and variations in normal knees. Bone Jt J. 2015;97–B(4):498–502. 10.1302/0301-620X.97B4.33740 25820888

[8] Delport H , Labey L , De Corte R , Innocenti B , Vander Sloten J , Bellemans J . Collateral ligament strains during knee joint laxity evaluation before and after TKA. Clin Biomech. 2013;28(7):777–782. 10.1016/j.clinbiomech.2013.06.006 23820027

[9] Freeman MAR , Pinskerova V . The movement of the normal tibio‐femoral joint. J Biomech. 2005;38(2):197–208. 10.1016/j.jbiomech.2004.02.006 15598446

[10] Graichen H , Avram GM , Zambianchi F , Graichen NM , Catani F , Lustig S , et al. Bony alignment decisions affect patient‐specific laxity phenotype patterns significantly, independent of the deformity. Knee Surg Sports Traumatol Arthrosc. 2025;33:3637–3645. 10.1002/ksa.12730 40583413

[11] Graichen H , Lekkreusuwan K , Eller K , Grau T , Hirschmann MT , Scior W . A single type of varus knee does not exist: morphotyping and gap analysis in varus OA. Knee Surg Sports Traumatol Arthrosc. 2022;30(8):2600–2608. 10.1007/s00167-021-06688-4 34414473

[12] Hamilton LD , Andreassen TE , Myers CA , Shelburne KB , Clary CW , Rullkoetter PJ . Knee pivot location in asymptomatic older adults. J Biomech. 2023;149: 1–20. 10.1016/j.jbiomech.2023.111487 PMC10080542 36868041

[13] Harfe D , Chuinard C , Espinoza L , Thomas K , Solomonow M . Elongation patterns of the collateral ligaments of the human knee. Clin Biomech. 1998;13(3):163–175. 10.1016/s0268-0033(97)00043-0 11415784

[14] Higinbotham SE , Ali AA , Liu X , Blackwood CA , Rullkoetter PJ , Blackwood CB , et al. Implant placement parameters are associated with post operative dynamic varus and valgus knee angle in robotic assisted TKA. Sci Rep. 2025;15:13143. 10.1038/s41598-025-98057-1 PMC12003682 40240515

[15] Hirschmann MT , Moser LB , Amsler F , Behrend H , Leclerq V , Hess S . Functional knee phenotypes: a novel classification for phenotyping the coronal lower limb alignment based on the native alignment in young non‐osteoarthritic patients. Knee Surg Sports Traumatol Arthrosc. 2019;27(5):1394–1402. 10.1007/s00167-019-05509-z 30976825

[16] Hodgeson SM , Soeno T , Mears SC , Stambough JB , Barnes CL , Stronach BM . The medial pivot design in total knee arthroplasty. Orthop Clin North Am. 2024;55(1):49–59. 10.1016/j.ocl.2023.06.007 37980103

[17] Hosseini Nasab SH , Smith CR , Schütz P , Postolka B , List R , Taylor WR . Elongation patterns of the collateral ligaments after total knee arthroplasty are dominated by the knee flexion angle. Front Bioeng Biotechnol. 2019;7:482970. 10.3389/fbioe.2019.00323 PMC6861521 31799245

[18] Hull ML . Coordinate system requirements to determine motions of the tibiofemoral joint free from kinematic crosstalk errors. J Biomech. 2020;109:1–8. 10.1016/j.jbiomech.2020.109928 32807309

[19] Indelli PF . The epidemic of alignment classifications in total knee arthroplasty forgives the kinematic of the human knee. J Exp Orthop. 2024;11(4):e70052. 10.1002/jeo2.70052 PMC11499622 39449827

[20] Karasavvidis T , Pagan Moldenhauer CA , Haddad FS , Hirschmann MT , Pagnano MW , Vigdorchik JM . Current concepts in alignment in total knee arthroplasty. J Arthroplast. 2023;38(7):S29–S37. 10.1016/j.arth.2023.01.060 36773657

[21] Kenanidis E , Milonakis N , Maslaris A , Tsiridis E . Robotic evaluation of articular laxity (REAL) classification: a new intraoperative knee soft‐tissue laxity classification using ROSA robotic software. Eur J Orthop Surg Traumatol orthopedie traumatologie. 2025;35(1):139. 10.1007/s00590-025-04265-w PMC11954700 40156716

[22] Koo S , Andriacchi TP . The knee joint center of rotation is predominantly on the lateral side during normal walking. J Biomech. 2008;41(6):1269–1273. 10.1016/j.jbiomech.2008.01.013 PMC2481385 18313060

[23] Lo J , Müller O , Dilger T , Wülker N , Wünschel M . Translational and rotational knee joint stability in anterior and posterior cruciate‐retaining knee arthroplasty. Knee. 2011;18(6):491–495. 10.1016/j.knee.2010.10.009 21074997

[24] MacDessi SJ , Griffiths‐Jones W , Harris IA , Bellemans J , Chen DB . Coronal plane alignment of the knee (CPAK) classification a new system for describing knee phenotypes. Bone Jt J. 2021;103 B(2):329–337. 10.1302/0301-620X.103B2.BJJ-2020-1050.R1 PMC7954147 33517740

[25] McClelland JA , Webster KE , Feller JA . Gait analysis of patients following total knee replacement: a systematic review. Knee. 2007;14(4):253–263. 10.1016/j.knee.2007.04.003 17531493

[26] Ophoff G , Bellemans J , Ascani D , Taylan O , Scheys L , Pandit H , et al. A navigation‐based analysis of native knee collateral ligament length change patterns in function of CPAK classification. Arch Orthop Trauma Surg. 2025;145(1):1–8. 10.1007/s00402-025-05984-9 40650751

[27] Ornetti P , Maillefert J‐F , Laroche D , Morisset C , Dougados M , Gossec L . Gait analysis as a quantifiable outcome measure in hip or knee osteoarthritis: a systematic review. Jt Bone Spine. 2010;77(5):421–425. 10.1016/j.jbspin.2009.12.009 20471899

[28] Qordja F , Valpiana P , Andriollo L , Rossi SMP , Salvi AG , Bocchino G , et al. The HKA axis varies significantly with knee motion: a robot‐assisted intraoperative evaluation during total knee arthroplasty supports the use of dynamic, not static, alignment classifications. J Exp Orthop. 2025;12(3):e70370. 10.1002/jeo2.70370 PMC12272511 40689101

[29] Rivière C , Iranpour F , Auvinet E , Howell S , Vendittoli P‐A , Cobb J , et al. Alignment options for total knee arthroplasty: a systematic review. Orthop Traumatol Surg Res. 2017;103(7):1047–1056. 10.1016/j.otsr.2017.07.010 28864235

[30] Sabatini L , Ascani D , Vezza D , Massè A , Cacciola G . Novel surgical technique for total knee arthroplasty integrating kinematic alignment and real‐time elongation of the ligaments using the NextAR system. J Pers Med. 2024;14(8):794–805. 10.3390/jpm14080794 PMC11355594 39201986

[31] Scholl LY , Hampp EL , Caba M , Azhar A , Hameed D , Dubin J , et al. Robotic‐assisted total knee arthroplasty technology provides a repeatable and reproducible method of assessing soft tissue balance. J Knee Surg. 2024;37(8):607–611. 10.1055/a-2232-7511 38113912

[32] Schütz P , Taylor WR , Postolka B , Fucentese SF , Koch PP , Freeman MAR , et al. Kinematic evaluation of the GMK Sphere implant during gait activities: a dynamic videofluoroscopy study. J Orthop Res. 2019;37(11):2337–2347. 10.1002/jor.24416 PMC6851890 31304995

[33] Singh V , Fiedler B , Huang S , Oh C , Karia RJ , Schwarzkopf R . Patient acceptable symptom state for the forgotten joint score in primary total knee arthroplasty. J Arthroplast. 2022;37(8):1557–1561. 10.1016/j.arth.2022.03.069 35346809

[34] Su S , He J , Wang R , Chen Z , Zhou F . The effectiveness of virtual reality, augmented reality, and mixed reality rehabilitation in total knee arthroplasty: a systematic review and meta‐analysis. J Arthroplast. 2024;39(3):582–590.e4. 10.1016/j.arth.2023.08.051 37598785

[35] Valpiana P , Salvi AG , Ghirardelli S , Bernardi M , Petralia G , Aloisi G , et al. Asymmetric gap balancing improves knee kinematic following primary total knee arthroplasty. Arthroplasty. 2024;6:29. 10.1186/s42836-024-00243-5 PMC11044442 38659002

[36] Verstraete MA , Meere PA , Salvadore G , Victor J , Walker PS . Contact forces in the tibiofemoral joint from soft tissue tensions: implications to soft tissue balancing in total knee arthroplasty. J Biomech. 2017;58:195–202. 10.1016/j.jbiomech.2017.05.008 28579262

[37] Viceconti M , Ascani D , Mazzà C . Pre‐operative prediction of soft tissue balancing in knee arthoplasty part 1: effect of surgical parameters during level walking. J Orthop Res. 2019;37(7):1537–1545. 10.1002/jor.24289 PMC6617758 30908694

[38] Victor J , Wong P , Witvrouw E , Sloten JV , Bellemans J . How isometric are the medial patellofemoral, superficial medial collateral, and lateral collateral ligaments of the knee? Am J Sports Med. 2009;37(10):2028–2036. 10.1177/0363546509337407 19589921

[39] Walker PS . Kinematics of the knee. In: Tria Jr., AJ , Scuderi GR , editors. The cruciate ligaments in total knee arthroplasty. Cham: Springer; 2024.

[40] Wirekoh J , Parody N , Meere PA . Functional knee apparatus for the evaluation of ligamentous tensions on contact loads. Knee. 2022;39:227–238. 10.1016/j.knee.2021.08.004 36257178

